# Prediction of Visual Field Progression in Myopic Normal Tension Glaucoma Using a Nomogram-Based Model

**DOI:** 10.3390/jcm15072709

**Published:** 2026-04-03

**Authors:** Ji Eun Song, Eun Ji Lee, Tae-Woo Kim

**Affiliations:** 1 Department of Ophthalmology, Kangwon National University Hospital, Kangwon National University College of Medicine, Chuncheon 24289, Republic of Korea; 2 Department of Ophthalmology, Seoul National University Bundang Hospital, Seoul National University College of Medicine, Seongnam 13620, Republic of Korea

**Keywords:** myopia, normal tension glaucoma, prediction, nomogram

## Abstract

**Background/Objectives**: This study aimed to develop a nomogram-based prediction tool to estimate visual field (VF) progression in patients with bilateral myopic normal-tension glaucoma (mNTG) by integrating key structural and vascular parameters. **Methods**: This retrospective cohort study included 150 eyes from 75 treatment-naïve patients with mNTG. All subjects were followed for at least five years with at least six reliable VF examinations. Key structural features, including the lamina cribrosa steepness index (LCSI) via enhanced-depth imaging optical coherence tomography (OCT) and choroidal microvascular dropout (cMvD) via OCT angiography (OCTA), were evaluated. VF progression was determined by event-based glaucoma progression analysis (GPA). To construct the predictive nomogram, clustered logistic regression with forward selection and 1000 bootstrap iterations was used to identify independent predictors. **Results**: Of the 150 eyes, 58 (38.7%) exhibited VF progression. Multivariable analysis identified steeper LCSI and the presence of parapapillary cMvD at baseline as significant independent predictors of progression. The resulting nomogram demonstrated excellent predictive accuracy, with an AUC of 0.922 and a C-index of approximately 0.92, indicating strong discriminative ability. **Conclusions**: This nomogram, incorporating structural (LCSI) and vascular (cMvD) markers, may offer a useful individualized tool for predicting VF progression in mNTG. This tool could assist in the early identification of high-risk patients and supports personalized treatment planning to optimize long-term visual outcomes.

## 1. Introduction

Myopia has long been recognized as a potential risk factor for the development and progression of glaucoma [1,2], a leading cause of irreversible blindness worldwide. Despite extensive research, the precise role of myopia in glaucoma progression remains controversial. Although some studies have reported an association between myopia and glaucoma progression [1,3,4], others have yielded conflicting results [5,6], leading to uncertainty in clinical management strategies. Furthermore, prior investigations into the relationship between optic nerve head (ONH) morphology—such as disc tilt or parapapillary atrophy (PPA)—and glaucomatous progression in myopic eyes have produced inconsistent results [7,8,9,10,11,12], further complicating our understanding of this multifaceted condition.

Understanding these morphological variations is crucial for guiding clinical management. Biomechanical changes within the lamina cribrosa (LC), the primary site of glaucomatous damage, have been implicated in ganglion cell axonal injury and subsequent optic neuropathy [13,14]. However, the relationship between LC deformation and glaucoma progression remains unclear, particularly in myopic eyes.

In addition to traditional ONH morphology, recent studies have highlighted the role of vascular factors in glaucoma progression. Parapapillary choroidal microvasculature dropout (cMvD) has emerged as a significant indicator of localized ischemia in the parapapillary region, reflecting compromised perfusion that contributes to retinal nerve fiber layer (RNFL) thinning and optic nerve vulnerability.

Despite advances in understanding the interplay between myopia and glaucoma, the factors associated with glaucoma progression in myopic patients remain insufficiently understood. Therefore, this study aimed to develop a nomogram-based prediction tool for visual field (VF) progression in patients with myopic normal-tension glaucoma (mNTG) by leveraging longitudinal data and incorporating key clinical parameters, including ONH morphology, LC curvature, and vascular characteristics. Ultimately, the goal of this tool is to enhance patient outcomes through individualized risk assessment and management strategies.

## 2. Materials and Methods

This study was approved by the Institutional Review Board of Seoul National University Bundang Hospital (B-2207-769-106) and adhered to the tenets of the Declaration of Helsinki.

### 2.1. Subjects

This retrospective study reviewed the medical records of patients diagnosed with normal-tension glaucoma (NTG) and myopia who visited our institution between January 2015 and January 2016. The baseline was defined as each patient’s first optical coherence tomography angiography (OCTA) performed in 2015, at which point all patients were treatment-naïve. Patients were included if they were treatment-naïve at baseline and had at least five years of follow-up. Anti-glaucoma treatment was initiated during follow-up when clinically meaningful progression was identified, primarily based on confirmed VF progression on serial event-based GPA together with overall clinical judgment. Prostaglandin analogues were used as first-line therapy in most cases. Each patient underwent a comprehensive ophthalmic examination, including best-corrected visual acuity, refraction, Goldmann applanation tonometry, slit-lamp examination, gonioscopy, dilated stereoscopic examination, color disc and red-free photography (EOS D60 digital camera; Canon, Utsunomiyashi, Japan), central corneal thickness (CCT) measurement (Orbscan II; Bausch & Lomb Surgical, Rochester, NY, USA), axial length (AL) measurement (IOL master 700; Carl Zeiss Meditec, Dublin, CA, USA), enhanced-depth imaging (EDI) spectral-domain OCT (SD-OCT; Spectralis OCT, Heidelberg Engineering, Heidelberg, Germany) scanning of optic disc, swept-source OCT angiography (OCTA; DRI OCT Triton, Topcon, Tokyo, Japan), and standard automated perimetry (Humphrey Field Analyzer II 750, 24-2, Swedish interactive threshold algorithm; Carl Zeiss Meditec).

The inclusion criteria were: diagnosis of NTG and myopia (spherical equivalent [SE] ≤ −0.5 diopters (D) and AL > 24.5 mm) at baseline examination, age over 20 years, best-corrected visual acuity of 20/40 or better, and at least five years of follow-up. NTG was defined as the presence of glaucomatous optic disc changes with corresponding abnormal VF loss, an open anterior chamber angle on gonioscopy, and maximum intraocular pressure (IOP) ≤ 21 mmHg without glaucoma medications. Patients with extreme myopia (SE < −10 D) were excluded due to potential structural retinal changes (e.g., lacquer cracks, posterior staphyloma) that could compromise the accuracy of measurements of myopic refractive error, AL, and even the RNFL. Additional exclusion criteria included history of ocular surgery other than cataract extraction, long-term steroid use, an identifiable cause of secondary glaucoma, or intraocular or neurological conditions that could affect VF testing.

### 2.2. Visual Field Examination and Progression Assessment

All participants underwent VF testing, and only reliable VF test results were included. A glaucomatous VF defect was defined as (1) an “outside normal limits” result on the glaucoma hemifield test, (2) three or more points with *p* < 1% on pattern deviation, or (3) a pattern standard deviation with *p* < 5% [15]. Abnormal findings were confirmed on two consecutive tests with fixation loss rate ≤ 20% and false positive/negative rates ≤ 25%. To calculate a reliable mean deviation (MD) slope, all patients included in the analysis had at least six reliable VF tests over at least 5 years of follow-up. The rate of change in MD was calculated by linear regression analysis using all reliable VF tests obtained from baseline to the final follow-up.

VF progression was assessed using event-based Glaucoma Progression Analysis software (GPA; version 4.2, Carl Zeiss Meditec Inc.). In this software, “likely progression” on event-based GPA software was defined as deterioration at the same three or more points across three consecutive follow-up tests [16].

### 2.3. Enhanced-Depth Imaging Optical Coherence Tomography of the Optic Disc

The ONH was imaged using SD-OCT with an EDI technique. The advantages of this technique for evaluating LC have been reported in previous studies [17,18]. Scanning was performed over a 10° × 15° rectangular area centered on the disc, consisting of approximately 70 horizontal B-scans spaced 30–40 µm apart. Each scan was averaged over 42 frames to optimize image clarity and patient cooperation [17].

ONH measurements included disc and PPA areas, with the latter subdivided into beta and gamma zones. Beta zone was defined by absence of retinal pigment epithelium, whereas gamma zone lacked Bruch’s membrane up to the inner border of the optic disc margin. All measurements were performed using in-built Spectralis OCT system software (Heidelberg Eye Explorer, version 6.16). Two masked evaluators (J.E.S. and E.J.L.) independently assessed all images, with discrepancies resolved by a third evaluator (T.W.K.).

### 2.4. OCTA and cMvD Assessment

The ONH and parapapillary regions were imaged using a commercially available swept-source OCTA device (DRI OCT Triton, Topcon) following established protocols [19]. A 4.5 mm × 4.5 mm cube scan centered on the optic disc was obtained, consisting of 320 clusters of four repeated B-scans. En face projections were created from volumetric scans to assess both the structural and vascular details across the segmented layers. Eyes with poor image quality or vascular signal artifacts were excluded.

cMvD was evaluated using en face OCTA images of the choroidal parapapillary layer generated using automated segmentation provided by the imaging device. The assessed region extended from Bruch’s membrane to 390 μm beneath it, encompassing the full thickness of the choroid and inner scleral layer.

cMvD was defined as a focal, sectoral loss of capillaries in which no microvascular network was visible on en face images, and the circumferential width of the dropout exceeded twice that of the adjacent visible juxtapapillary microvessels [19,20]. In cases where large retinal vessels were present, they were included in the cMvD measurement only if the dropout extended beyond the vessel borders; otherwise, they were excluded [21]. The presence and area of cMvD were independently assessed by two blinded glaucoma specialists (J.E.S. and E.J.L.), with any disagreements resolved by a third evaluator (T.W.K.).

### 2.5. Lamina Cribrosa Steepness Index (LCSI) Measurement

**Figure 1:**
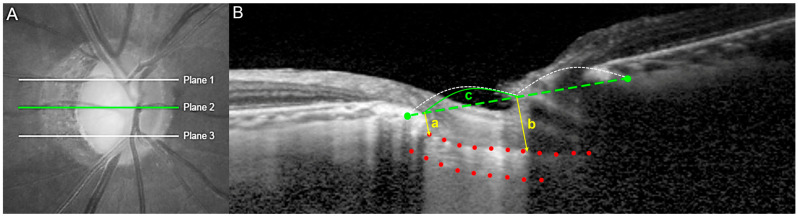
Measurement of the lamina cribrosa (LC) steepness index (LCSI). (**A**) Disc photograph showing three horizontal lines (planes 1, 2, and 3) across which LCSI measurements were taken. (**B**) Optical coherence tomography (OCT) B-scan image obtained at plane 2 as shown in (**A**). A horizontal reference line (green dotted line) was drawn between two green points marking the anterior scleral canal opening (ASO). Vertical distances were measured from this line to the anterior LC border (red dots): (a) at the temporal insertion site and (b) at the center of the reference line. LCSI was calculated as (b − a) × 100/c, where c represents the horizontal distance between the two ASO points.

The LCSI was assessed using horizontal B-scan images obtained with EDI OCT, optimized for LC surface visualization. The same scans used to calculate the lamina cribrosa curvature index (LCCI) were used to derive the LCSI [22], which reflects the inclination of the LC relative to the anterior scleral canal opening (ASO) plane. The LCSI measurement method is illustrated in Figure 1.

The LCSI was measured in three planes for each eye: the superior midperiphery (plane 1), mid-horizontal region (plane 2), and inferior midperiphery (plane 3) (Figure 1A). On each B-scan, a reference line was drawn connecting two points of the ASO. Two vertical distances were then measured: one from the reference line to the temporal insertion site of the LC (a), and another from the reference line to the anterior LC border at the center of the reference line (b). The LCSI was calculated using the formula (b − a) × 100/c, where c represents the horizontal distance between these two points along the ASO plane.

The mean LCSI was calculated by averaging the measurements obtained from the three planes. All measurements were performed using the manual caliper tool provided in the Heidelberg software (version 1.10.4, Heidelberg Engineering). Two independent observers (J.E.S. and E.J.L.), blinded to the patients’ clinical information, conducted the measurements. Their average values were used for primary analyses.

### 2.6. Data Analysis

Simple and multiple logistic regression analyses were conducted to develop a predictive model for VF progression in patients with mNTG. Variable selection was performed using a forward selection approach with bootstrapping (1000 iterations), and variables that were consistently selected across iterations were included in the final model.

Given that both eyes of some patients were included, a clustered logistic regression model was employed to adjust for intrasubject correlation. Continuous variables were categorized based on median values. A nomogram was constructed based on the final predictive model, which included the variables consistently selected through forward selection and bootstrapping to estimate the individual risk of glaucoma progression. Internal validation of the model was performed using the bootstrap resampling method (1000 iterations) to assess model stability. The predictive performance of the model was evaluated using both the C-index and the receiver operating characteristic (ROC) curve.

All statistical analyses were conducted using IBM SPSS Statistics software (version 22.0; SPSS, Chicago, IL, USA) and R software (version 4.1.1; R Project for Statistical Computing). The Multivariate Imputation by Chained Equations (mice) package (version 3.16.0) was used for multiple imputations, and the regression Modeling strategies (rms) package (version 6.8-0) was employed for regression modeling.

## 3. Results

### 3.1. Patient Characteristics and Baseline Demographics

Medical records of 276 patients with bilateral mNTG who visited Seoul National University Bundang Hospital between January 2015 and January 2016 were reviewed by a glaucoma specialist (J.E.S). Of these, 105 patients were treatment-naive at baseline. After excluding patients due to poor image quality and low-test reliability (*n* = 25), and high myopia with myopic retinal degeneration (*n* = 5), 150 eyes from 75 treatment-naïve patients were ultimately included. All patients underwent at least six reliable VF examinations and were followed up for at least five years. To ensure longitudinal consistency and reproducibility, only patients evaluated using the exact same hardware and software versions, with serial OCT and baseline OCTA images of sufficient quality for reliable analysis, were included.

Among the 150 eyes, 92 (61.3%) eyes were classified as non-progressive and 58 (38.7%) as progressive. Baseline characteristics of the two groups are presented in Table 1. No significant differences were observed in sex, family history of glaucoma, migraine status, baseline spherical equivalent, AL, central corneal thickness, disc tilt ratio, PPA area, disc area, baseline IOP, baseline global RNFLT, or baseline MD and PSD on VF testing between the two groups. However, DH was significantly more frequent in the progressive group than in the non-progressive group (24.1% vs. 2.2%, *p* < 0.001).

### 3.2. Risk Factors Associated with VF Progression

**Figure 2:**
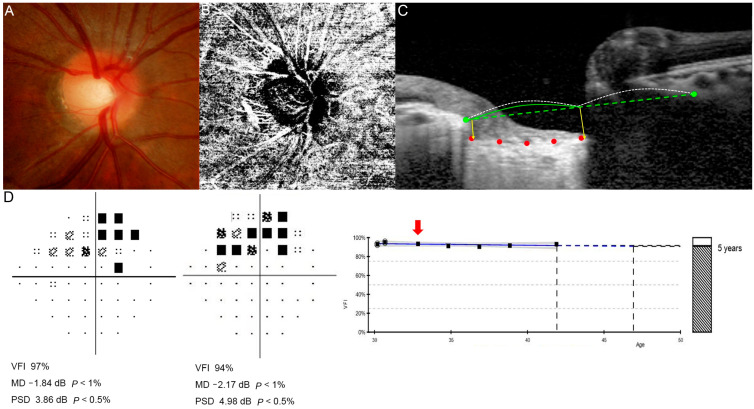
Representative case of a 30-year-old woman with non-progressive myopic normal-tension glaucoma (mNTG) who was not treated with glaucoma medication. (**A**) Fundus photography, (**B**) optical coherence tomography angiography (OCTA), and (**C**) lamina cribrosa (LC) image at the initial visit, and (**D**) visual field (VF) examination at the initial and 13-year follow-up. In panel (**C**), green points indicate the anterior scleral canal opening (ASO) connected by a green dashed reference line. Red dots mark the anterior lamina cribrosa (LC) border. Yellow vertical lines represent distances *a* and *b*, while the white dotted curve indicates the mid-point of the reference line. In panel (**D**), the red arrow indicates the baseline examination among the series of follow-up VFs. No significant VF progression was observed over 12 years (GPA). The lamina cribrosa steepness index (LCSI) was 13.90, and the VF mean deviation (MD) changed from −1.84 dB at baseline to −2.17 dB at follow-up without treatment.

**Figure 3:**
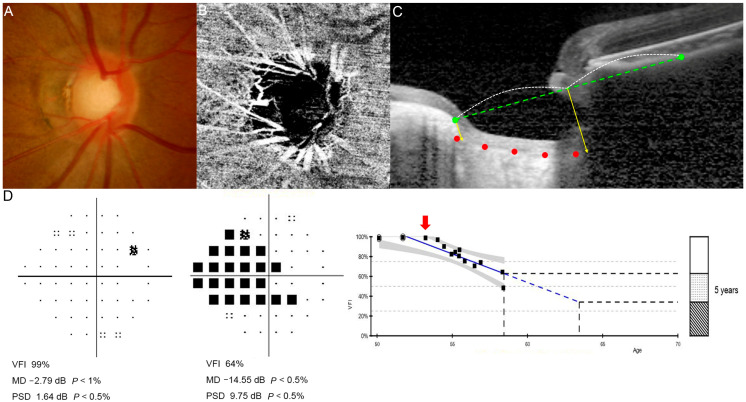
Representative case of a 50-year-old woman with progressive myopic normal-tension glaucoma (mNTG) who was initially untreated. (**A**) Fundus photography, (**B**) optical coherence tomography angiography (OCTA), and (**C**) lamina cribrosa (LC) image at the initial visit, and (**D**) visual field (VF) examination at the initial and 9-year follow-up. In panel (**C**), green points indicate the anterior scleral canal opening (ASO) connected by a green dashed reference line. Red dots mark the anterior lamina cribrosa (LC) border. Yellow vertical lines represent distances *a* and *b*, while the white dotted curve indicates the mid-point of the reference line. In panel (**D**), the red arrow indicates the baseline examination among the series of follow-up VFs. As progression occurred over time, glaucoma medication was initiated. The mean lamina cribrosa steepness index (LCSI) of the right eye was 39.88, and the VF mean deviation (MD) deteriorated from −2.79 dB at the initial visit to −14.55 dB at follow-up.

Figure 2 and Figure 3 illustrate representative cases of patients with myopic glaucomatous eyes. These include data obtained prior to the introduction of OCTA in 2015, with an arrow indicating the baseline OCTA time point in each GPA. In Figure 2, a patient with a relatively low LCSI and no cMvD exhibited no VF progression over 13 years without any ocular hypotensive treatment. In contrast, Figure 3 shows a patient with a steep LCSI and extensive cMvD who showed rapid VF deterioration despite aggressive glaucoma treatment.

Among eyes with VF progression, baseline characteristics were additionally compared between the early and late progression groups (Supplementary Table S1). Eyes with early progression showed a higher baseline IOP and a greater frequency of DH than those with late progression, while other baseline parameters were not significantly different.

### 3.3. Development and Validation of a Predictive Nomogram

**Figure 4:**
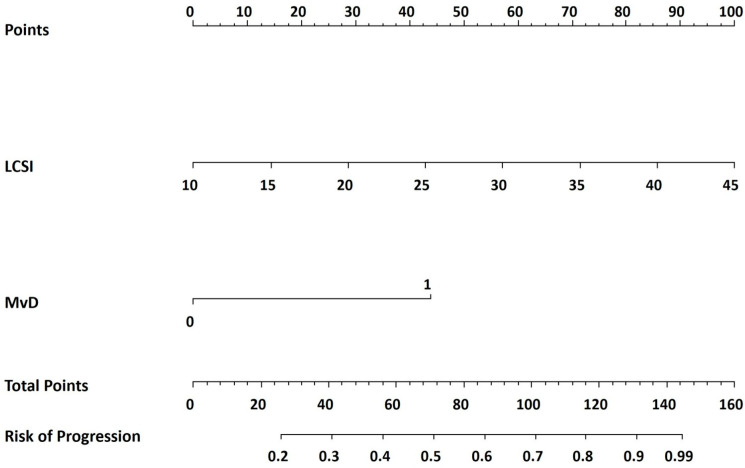
Nomogram for predicting visual field progression in patients with myopic normal-tension glaucoma (mNTG). For each predictor, a vertical line is drawn upward to the “Points” scale to determine the corresponding score. The individual scores are then summed to obtain the “Total Points,” which corresponds to the predicted probability of visual field progression on the bottom scale. Higher total points indicate a higher predicted risk of progression.

**Figure 5:**
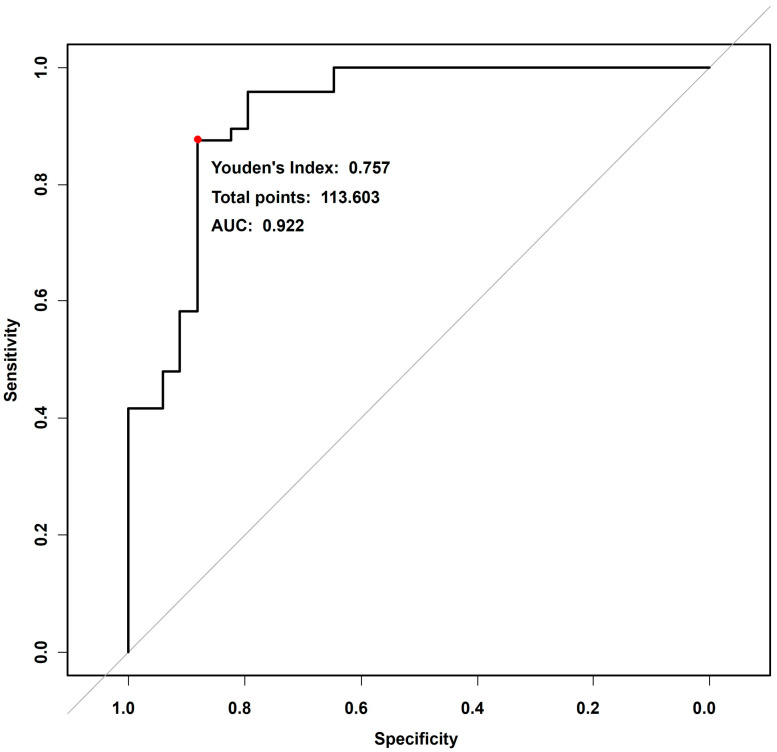
Receiver operating characteristic (ROC) curve showing the predictive performance of the nomogram. The area under the curve (AUC) was 0.922, indicating excellent discrimination between progression and non-progression. The red dot on the curve indicates the optimal cut-off point determined by the maximum Youden’s Index (0.757), representing the best balance between sensitivity and specificity.

Using the independent and significant risk factors identified through multiple logistic regression, we developed a predictive model for VF progression and presented it as a nomogram (Figure 4). In the nomogram, the LCSI contributed the largest proportion of cases, followed by parapapillary cMvD. The total score was calculated by summing the points assigned to each risk factor, which was then used to estimate the probability of glaucoma progression. To illustrate the practical use of the nomogram, we applied it to the representative cases shown in Figure 2 and Figure 3. The non-progression case in Figure 2 (LCSI, 13.90; no MvD) corresponded to approximately 11 total points, indicating a predicted progression risk of less than 10%. In contrast, the progression case in Figure 3 (LCSI, 39.88; MvD present) corresponded to approximately 129 total points, indicating a predicted risk of more than 90%. ROC curve analysis confirmed the high predictive accuracy of the nomogram. As shown in Figure 5, the AUC was 0.922, indicating excellent discriminative ability between progression and non-progression groups. This robust AUC supports the utility of the nomogram as a reliable predictive tool.

## 4. Discussion

This study developed and internally validated a nomogram-based model to predict VF progression in treatment-naïve eyes with mNTG. Our findings identified two key baseline factors, LCSI and cMvD, that together provided strong prognostic value. The resulting nomogram offers a practical and individualized graphical tool to estimate the risk of glaucoma progression, supporting tailored clinical decision-making.

In patients with myopic eyes, diagnosing and monitoring glaucoma progression can be challenging due to structural changes around the ONH, including large disc area and tilt, enlarged PPA, LC thinning, and a shallow optic cup resulting from axial elongation [7,8,9,23]. These features may obscure glaucomatous changes or be mistaken for glaucomatous damage [22,24,25]. In our previous work, we demonstrated that the LCCI is a valuable parameter for evaluating glaucoma in such patients [24,26]. However, assessing LC morphology in the nasal disc region is often difficult in myopic eyes. To overcome this, we developed the LCSI, a novel parameter that quantifies LC curvature using only the temporal region. In our previous study, the LCSI was strongly correlated with LCCI, suggesting its reliability as a surrogate metric in myopic populations [22,24].

The presence of parapapillary cMvD was also identified as an independent predictor of disease progression, highlighting the role of vascular factors in NTG. Previous studies have shown associations between cMvD and glaucomatous damage in both NTG and high-tension glaucoma [27,28,29]. Our findings reinforce the idea that cMvD is not merely a secondary phenomenon but likely reflects localized ischemia contributing to RNFL loss. In our cohort, eyes with more extensive dropouts affecting both superior and inferior regions were more prone to rapid progression. These findings are consistent with those of Kim et al. [28], who reported that progressive cMvD enlargement correlates with accelerated RNFL thinning. Moreover, the topographic correspondence between cMvD and focal LC damage observed in another study [30] suggests that chronic choroidal hypoperfusion may underlie both vascular and structural deterioration, leading to VF loss.

Although DH was significantly more frequent in the progression group, it was not retained as an independent predictive factor in the multivariate model. This may be due to the stronger predictive power of the LCSI and cMvD, which could overshadow the impact of DH in the multivariate model. Although DH showed a significant difference between the groups, its relative importance diminished when combined with these more robust and stable baseline predictors. Nonetheless, the occurrence of DH remains an important clinical sign of high risk [31,32]. Future models may benefit from incorporating DH as a time-dependent covariate during follow-up to enhance long-term risk prediction.

Han et al. [33] previously reported a slower VF progression rate (−0.23 dB/year) in untreated myopic NTG compared to our cohort’s average progression of −0.964 dB/year. This discrepancy likely reflects differences in cohort composition: their study included non-progressors who required no treatment, whereas ours focused on fast progressors who required active intervention. Additionally, the patients in our study have a higher degree of myopia (mean SE: −4.6 D) compared to the previous report (mean SE: −3.5 D). These findings are consistent with prior research indicating that moderate-to-severe myopia is a significant risk factor for glaucoma progression [34,35].

The clinical utility of our nomogram lies in its ability to simplify complex risk profiles into clinically actionable predictions. By incorporating both LCSI and cMvD, clinicians can estimate the likelihood of significant VF deterioration over a five-year period. For example, a patient with a steep LCSI and detectable cMvD may have a predicted risk exceeding 80%, suggesting the need for early and aggressive management, even with IOP in the normal range. In contrast, a patient with a relatively flat LCSI and no cMvD may be managed conservatively, thereby avoiding unnecessary interventions. The model’s high C-index of approximately 0.92 affirms its excellent discriminative performance.

Despite these promising results, several limitations should be considered. First, the study’s retrospective design and relatively small sample size limit generalizability. Patients with glaucoma who did not progress may have discontinued follow-up, introducing selection bias. Second, patients were enrolled at varying stages of mNTG, and some with advanced but stable disease may have progressed earlier in the disease course. A prospective study with uniform baseline staging is needed. In addition, anti-glaucoma treatment was initiated during follow-up in eyes with progression, and subsequent IOP reduction may have influenced later progression. Because treatment effects were not separately analyzed, and longitudinal factors such as IOP fluctuation during follow-up were not included in the model, these issues should be considered when interpreting the results. Furthermore, the primary objective of this study was to develop a baseline-entry tool for risk stratification at the initial visit using only parameters available at baseline. Third, the study population consisted entirely of Korean patients. As LC structure and response to IOP may vary across ethnicities [36,37], further longitudinal studies involving different ethnic groups are required. Fourth, the model was not externally validated using an independent cohort. Although robust internal validation using 1000 bootstrap iterations demonstrated good stability (C-index, approximately 0.92), bootstrap-based validation cannot substitute for true external validation. Therefore, the proposed nomogram should be regarded as a preliminary risk-stratification tool, and its use as a stand-alone model in routine clinical practice cannot yet be recommended. Accordingly, no fixed treatment cutoff was proposed, and the nomogram should instead be interpreted as a tool for relative risk stratification. Finally, OCTA was performed only at baseline, and no follow-up examinations were conducted. Without longitudinal OCTA imaging, we could not assess changes in microvasculature over time. Future studies should incorporate repeated OCTA to better understand dynamic vascular changes and their relationship to glaucoma progression.

## 5. Conclusions

In conclusion, this study suggests that in mNTG, where conventional structural markers may be affected by myopic changes, integrating the LCSI and cMvD may improve the prediction of VF progression. The proposed nomogram may help identify eyes at relatively higher risk of progression and may serve as a preliminary tool for individualized risk stratification. However, further external validation is required before it can be recommended for routine clinical application or treatment guidance.

## Figures and Tables

**Table 1:** Clinical characteristics of the study participants.

	Non-Progression (*n* = 92)	Progression (*n* = 58)	*p* Value *
Age, years	44.26 ± 10.68	42.55 ± 11.971	0.364
Female, *n* (%)	40 (43.48)	31 (53.45)	0.236
Family History of Glaucoma, *n* (%)	16 (17.39)	10 (17.24)	0.981
Migraine, *n* (%)	8 (8.70)	12 (20.69)	0.053
SE, D	−4.32 ± 3.35	−5.04 ± 3.49	0.216
AXL, mm	26.31 ± 1.55	26.16 ± 1.55	0.581
CCT, µm	537.54 ± 35.03	534.29 ± 31.33	0.590
Optic disc characteristics			
Disc hemorrhage, *n* (%)	2 (2.17)	14 (24.14)	**<0.001**
Disc tilt ratio	1.31 ± 0.22	1.32 ± 0.19	0.817
Area of beta-zone PPA, mm^2^	0.97 ± 0.50	1.11 ± 0.78	0.078
Area of gamma-zone PPA, mm^2^	0.78 ± 0.61	0.85 ± 0.71	0.194
Area of disc, mm^2^	2.21 ± 0.55	2.21 ± 0.66	0.997
Baseline IOP, mmHg	13.17 ± 2.08	13.82 ± 1.57	0.127
Baseline global RNFLT, μm	71.52 ± 9.26	69.03 ± 11.73	0.248
VF examination			
Baseline MD, dB	−4.76 ± 5.18	−5.99 ± 6.21	0.563
Baseline PSD, dB	4.39 ± 3.90	5.54 ± 4.24	0.920
Lamina Cribrosa Steepness Index	17.96 ± 3.64	23.97 ± 6.36	**<0.001**
Microvascular Dropout	15 (16.30)	50 (86.21)	**<0.001**
Systolic BP, mmHg	121.83 ± 13.32	124.65 ± 16.11	0.276
Diastolic BP, mmHg	73.94 ± 10.81	75.17 ± 12.25	0.544
Rate of progression in MD per year, dB	0.12 ± 0.25	−0.964 ± 0.60	**<0.001**
Follow-up, years (range)	7.76 ± 2.65 (5–10)	7.97 ± 2.61 (5–10)	0.644

Data are presented as mean ± standard deviation or *n* (%). * Independent *t*-test; *p* values in boldface indicate statistical significance. Abbreviations: SE = spherical equivalent; AXL = axial length; CCT = central corneal thickness; PPA = parapapillary atrophy; IOP = intraocular pressure; RNFLT = retinal nerve fiber layer thickness; VF = visual field; MD = mean deviation; PSD = pattern standard deviation; D = diopters; dB = decibels; BP = blood pressure.

## Data Availability

The data presented in this study are available on request from the corresponding author due to patients’ privacy and ethical considerations.

## Supplementary Materials

The following supporting information can be downloaded at: https://www.mdpi.com/article/10.3390/jcm15072709/s1, Table S1: Comparison of baseline demographic and clinical characteristics between the early and late progression groups.

## Abbreviations

The following abbreviations are used in this manuscript:

VF	Visual Field
mNTG	Myopic Normal Tension Glaucoma
ROC	Receiver Operating Characteristic
AUC	Area Under the Receiver Operating Characteristic Curve
IOP	Intraocular Pressure
DH	Disc Hemorrhage
OCT	Optical Coherence Tomography
OCTA	Optical Coherence Tomography Angiography
cMvD	Choroidal Microvascular Dropout
GPA	Glaucoma Progression Analysis
EDI	Enhanced-Depth Imaging
SD-OCT	Spectral-Domain Optical Coherence Tomography
SAP	Standard Automated Perimetry
ONH	Optic Nerve Head
C-index	Concordance Index
PPA	Parapapillary Atrophy
LC	Lamina Cribrosa
LCSI	Lamina Cribrosa Steepness Index
AL	Axial Length
CCT	Central Corneal Thickness
MD	Mean Deviation
PSD	Pattern Standard Deviation
ASO	Anterior Scleral Canal Opening
LCCI	Lamina Cribrosa Curvature Index
Mice	Multivariate Imputation by Chained Equations
Rms	Regression Modeling Strategies
