# Supplementary material for: Prediction of Visual Field Progression in Myopic Normal Tension Glaucoma Using a Nomogram-Based Model
Source: J Clin Med. 2026 Apr 3;15(7):2709. doi: 10.3390/jcm15072709 (PMC13073007; doi:10.3390/jcm15072709)
Supplement: Supplementary file 1 [file jcm-15-02709-s001.zip › jcm-4176291-supplementary.pdf]

Supplementary Table S1. Comparison of baseline demographic and clinical characteristics between the early and late progression groups.

	Early Progressor (n = 32)	Late Progressor (n = 26)	*P* value*
Age, years	42.09 ± 12.04	43.12 ± 12.09	0.750
Female, n (%)	17 (53.13)	10 (38.46)	0.273
SE, D	-5.37 ± 3.94	-4.63 ± 2.85	0.425
AXL, mm	26.24 ± 1.32	26.07 ± 1.82	0.687
CCT, μm	536.10 ± 30.82	532.19 ± 32.40	0.646
Optic disc characteristics			
Disc hemorrhage, n (%)	11 (34.38)	3 (11.54)	**0.037**
Disc tilt ratio	1.34 ± 0.21	1.28 ± 0.16	0.218
Area of beta-zone PPA, mm^2	1.25 ± 0.89	1.10 ± 0.62	0.484
Area of gamma-zone PPA, mm^2	1.00 ± 0.68	0.75 ± 0.61	0.056
Area of disc, mm^2	2.11 ± 0.71	2.36 ± 0.55	0.138
Baseline IOP, mmHg	14.47 ± 1.55	12.69 ± 1.89	**<0.001**
Baseline global RNFLT, μm	69.69 ± 12.03	71.40 ± 10.73	0.387
VF examination			
Baseline MD, dB	-6.51 ± 6.02	-4.75 ± 4.29	0.122
Baseline PSD, dB	6.43 ± 4.60	6.27 ± 3.81	0.886
Lamina Cribrosa Steepness Index	24.77 ± 7.21	22.89 ± 4.94	0.279
Microvascular Dropout	30 (93.75)	20 (76.92)	0.250
Systolic BP, mmHg	125.71 ± 16.73	123.50 ± 15.67	0.619
Diastolic BP, mmHg	76.00 ± 13.23	74.27 ± 11.29	0.609

Data are presented as mean ± standard deviation or n (%)
*Independent t-test; *P* values in boldface indicate statistical significance.
Abbreviations: SE = spherical equivalent; AXL = axial length; CCT = central corneal thickness; PPA = parapapillary atrophy; IOP = intraocular pressure; RNFLT = retinal nerve fiber layer thickness; VF = visual field; MD = mean deviation; PSD = pattern standard deviation; D = diopters; dB = decibels; BP = blood pressure
